# Fast and simple method for screening of single-stranded DNA breaking photosensitizers using graphene oxide

**DOI:** 10.1186/s40580-018-0160-3

**Published:** 2018-10-22

**Authors:** Joong Hyun Kim, Hyun Jin Kim

**Affiliations:** Medical Device Development Center, Daegu-Gyeongbuk Medical Innovation Foundation, Daegu, 41061 South Korea

**Keywords:** Graphene oxide, Single-stranded DNA break, Skin cancer, Phototoxic drug

## Abstract

A fast and simple method for screening of ssDNA-breaking photosensitizers was developed using graphene oxide. The ultraviolet light-induced DNA breaks are one of the most harmful DNA damages and cause skin cancer if they are left unrepaired. Since graphene oxide showed relatively strong affinity to the broken DNA than intact DNA, and it quenched fluorescence of the DNA labeling dye effectively, the degree of ultraviolet light-induced broken DNAs could be analyzed by measuring decreased fluorescence after mixing the DNA with graphene oxide. The decrease of fluorescence was highly correlated with the ultraviolet light-irradiating time and concentration of the added drugs. As a result, it was possible to evaluate the efficacy of different ssDNA-breaking photosensitizers in a high-throughput manner. However, conventional methods for the damaged-DNA analysis are time-consuming and require additional manipulations such as purification, radio-labeling, enzymatic digestion, or chemical modification of DNA. The phototoxicity of five drugs such as benzophenone, ketoprofen, indomethacin, naproxen, and norfloxacin was tested using the proposed method. The ssDNA-breaking efficiency of the drugs was well matched with reported efficiency of the tested drugs. In contrast to naked gold nanoparticles, graphene oxide is stably dispersed in the presence of salt, the phototoxicity of the drugs could be successfully tested at a physiological condition using the graphene oxide based method.

## Introduction

In countries with a white population, the most common cancer is skin cancers. Every 10–20 years, incidence of melanoma has doubled in these countries. In 2007, more than 84,500 people in UK was diagnosed with non-melanoma skin cancer [1]. It was reported that about two-thirds of Australian before 70 years old could have skin cancer at least once for their life [2]. Unrepaired photolesions which are formed by ultraviolet (UV) light irradiation is considered as main cause of the skin cancer. The mutated photolesions such as bipyrimidine dimers, apurine sites and strand breaks are produced by adsorption of UV light [3, 4]. Among them, bipyrimidine dimers have the highest frequency than others. However, the other UV light-induced mutants formed to a lesser extent can be more harmful than bipyrimidine dimers [5]. In addition, it is known that occurrence of these types of cellular DNA damages are increased by several drugs due to photosensitization [5–9]. Because of  the phototoxicity of the photosensitizers, FDA recommends to provide the photosensitizing effects of drug substances. Comet assay [10] and Ames test [11] are representative in vitro genotoxicity tests as standard testing. Beside such cell-based methods, acelluar assays using naked DNA, such as immunological assays [12], gel electrophoresis [13], chromatic assays [14], atomic force microscopy [15] and electrochemical sensor-based assays [16], have been also developed to analyze the UV light-induced DNA mutations. However, these approaches are still limited to the high-throughput screening of compounds for phototoxicity because of their dependence on expensive instruments and additional manipulations such as purification, radiolabeling, enzymatic digestion, or chemical modification of the DNA for sensitive and accurate information. In the pharmaceutical industry there have been growing demands for a simplified method to screen for phototoxic chemical compounds in a parallel way, especially a method that can be implemented in an automated system. In previous, a gold nanoparticle (AuNP) based simple colorimetric method to detect the single-stranded (ss)DNA breaks (SDBs) and to screen photosensitizing drugs were reported [17]. However, the AuNP based colorimetric methods are not applicable to a salt containing physiological condition because AuNPs are easily aggregated by salt. However, the colorimetric method may not detect the phenomena under physiological conditions. In contrast, salt-stable dispersion of graphene oxide (GO) could allow its application to detect SDBs in the physiological condition. Due to the selective affinity to ssDNA, and highly effective fluorescence quenching ability, GO has been applied to detect various targets such as heavy metals, small molecules and nucleic acids [18]. Previously, a GO based method to detect DNA photodimers and screen dimer-photosensitizing drugs was reported [19]. However, it is not yet reported if GO could be applied to detect the SDBs and screen photosensitizing drugs. Therefore, in this study, GO was first applied to detect the SDBs and evaluate the photosensitizing effect of drugs under a physiological condition.

## Experimental

All other chemicals except for DNA were purchased from Sigma-Aldrich and used without further purification. The sequence of the DNA used for the detection of SDBs (5′-Texas reds^®^-GGAAGGTGTGGAAGG-3′) was purchased from Bioneer (Daejeon, Korea). Because the formation of pyrimidine photodimers will interfere with the interaction between ssDNA and GO, special care for designing DNA sequence that included only purines for the specific detection of SDBs was needed [17]. ssDNA was added in a quartz cuvette or a well plate followed by mixing with each drug in 18 µl of 1xPBS (pH 7.4). The DNA sample (100 nM) in the sealed quartz cuvette was irradiated under 25 cm of a UV lamp (15 W, 10,600 µW/cm^2^ at 312 nm), which was top of a dark box (35 cm × 35 cm × 30 cm) [17]. The irradiated energy to DNA was adjusted by varying the UV irradiation time. For the detection of SDBs, the UV light-irradiated DNA was mixed with GO (2 µg/ml) in PBS for 10 min. For the parallel screening of the photosensitizing drugs, DNA samples were mixed with 20 µM of each drug and irradiated under the UV lamp for 20 min. After 10 min of mixing with GO, fluorescence of the UV light-irradiated samples were recorded using a microplate reader (Infinite M200 Pro, Tecan, Switzerland) with excitation at 560 nm.

## Results and discussion

**Fig. 1:**
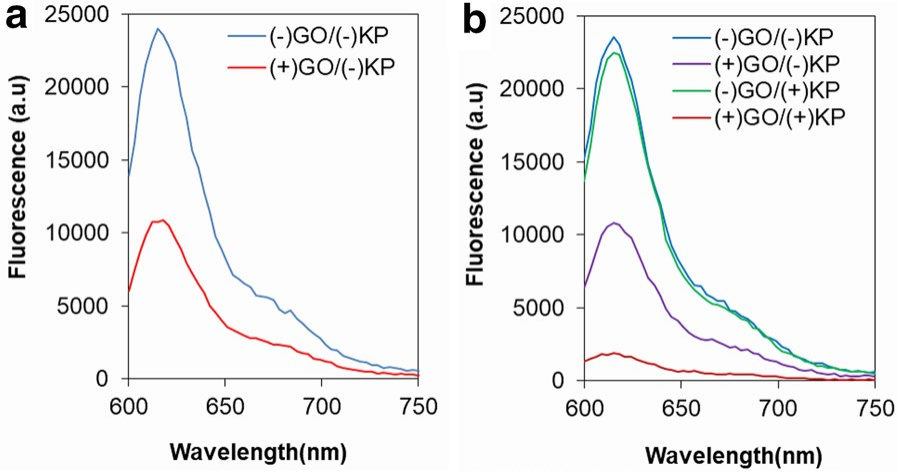
Fluorescence spectra of ssDNA before **a** and after **b** irradiation of UVB light in the absence (−) and presence of (+) GO and KP

Figure 1 shows recorded spectra of fluorescence of ssDNAs before and after mixing with GO depending on the UV irradiation and presence of ketoprofen (KP), one of the representative ssDNA-breaking photosensitizers [16]. Generally, fluorescence of the dye labeled ssDNA is effectively quenched by GO because of strong adsorption of ssDNA on GO and effective energy transfer to GO [18, 20]. As shown in Fig. 1, decrease of fluorescence (DOF) from ssDNA by GO was also observed in this study but there was additional fluorescence reduction by UV irradiation in the presence of KP. The additional DOF from the UV-irradiated DNA in the presence of KP was opposite of anticipation. In the previous colorimetric method, broken DNA under UV irradiation in the presence of KP could not stabilize AuNPs to prevent salt-induced aggregation [17]. Decreased affinity of the broken DNA was attributed to the aggregation of AuNPs. Similar aggregation of AuNPs mixed with the UV-irradiated DNA in the presence of KP used in this study was observed (Data not shown). No significant difference of fluorescence of the ssDNA before and after UV irradiation in the absence of GO indicates that UV irradiation did not affect the fluorescence of the labeling dye. However, DOF of the UV-irradiated DNA in the presence of KP indicates increased adsorption of DNA on GO. It was reported that shorter DNAs were adsorbed more rapidly and bound more tightly to GO [20]. The rapid adsorption of short DNA on AuNP was also reported while the shorter DNA was rapidly desorbed from AuNPs [20, 21]. When enough time was given for mixing of AuNPs with ssDNAs, AuNPs mixed with shorter DNA were more easily aggregated [21]. The difference of desorption kinetics of ssDNA on GO and AuNPs might result in DOF opposite of the expectation. In contrast, other reports showed less adsorption of shorter DNA on GO [22, 23]. Since the binding affinity of DNA on GO is function of various factors such as base compositions of DNA, salt concentration, size and concentration of GO, pH, temperature and so on [20]. More systematic study for the physical interactions between DNA and GO including all the variables should be investigated.

**Fig. 2:**
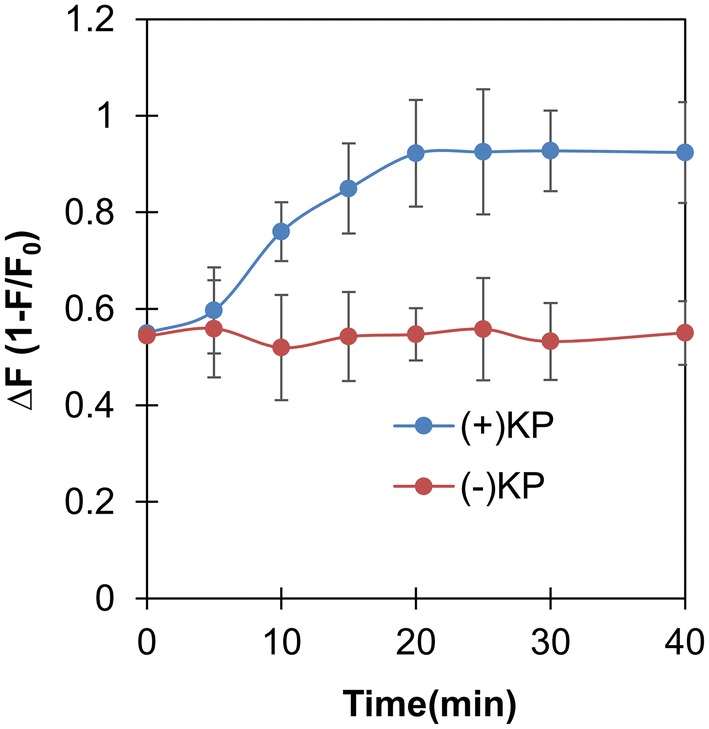
Time-dependent decrease of fluorescence in the mixture of GO and UVB-irradiated DNA at 618 nm compared to intact DNA. GO was mixed with ssDNA that had been irradiated with UVB light for various time. F and F_0_ is fluorescent of the mixture and intact DNA without GO respectively

Next, SDB depending on the irradiated energy was investigated by varying the UV-irradiating time and estimating DOF of the tested DNA comparing the fluorescence in the absence of GO. As shown in Fig. 2, the DOF of the UV-irradiated DNA-GO mixture increased proportionally to the UV-irradiating time and was almost maximized at 20 min. There was no change of DOF in DNA-GO mixture even after 20 min of the irradiation with UV light in the absence of KP. It is known that KP could lead to not only SDBs but also the formation of the photodimers between adjacent bases in a same strand DNA through triplet–triplet energy transfer in response to UV irradiation [6]. The formation of photosensitized dimer is highly dependent on the DNA bases. Pyrimidine dimers occur at the highest frequency [13]. However, it was reported that purine dimers formed between two adenines and between adjacent purines and pyrimidines [24]. Previously, it was reported that formation of dimers within DNA increased affinity of GO to the mutated DNA [19]. No significant changes in DOF of the GO-DNA mixtures in the absence of KP even after 40 min of irradiation with UV light confirmed no noticeable formation of purine dimers.

**Fig. 3:**
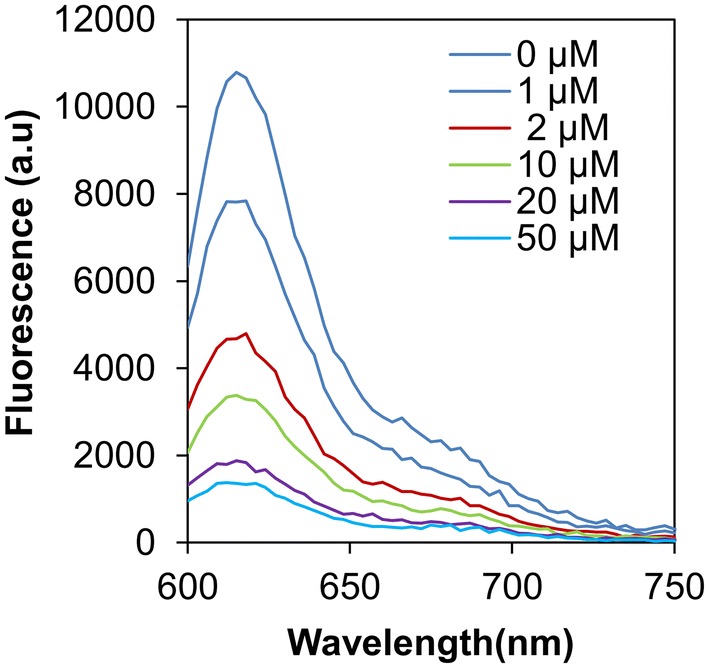
Fluorescence spectra of GO-DNA mixture. DNA was irradiated by UVB light for 20 min in various concentration of KP

In addition, dependence of SDB on the concentration of the photosensitizer was also studied. Figure 3 shows that the measured fluorescence of the GO-DNA mixtures decreased as the added KP to DNA increased. Rate of the DOF at KP higher than 20 µM became slow. The DOF of the mixtures proportional to the concentration of KP implies that the numbers of broken strands of DNA under UV irradiation also increased proportionally to the concentration of KP.

**Fig. 4:**
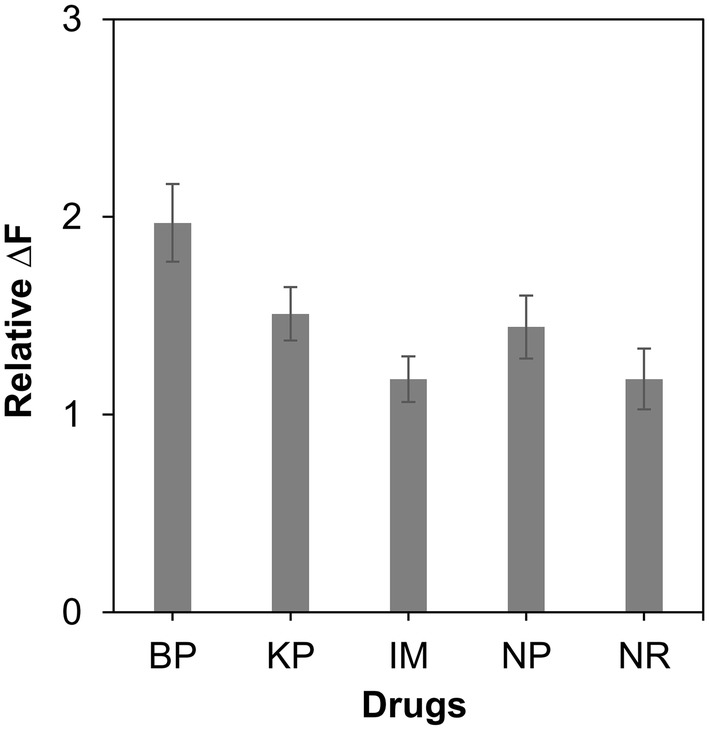
Parallel screening of SDB-photosensitizing drugs. After UVB irradiation of ssDNA in the presence of various drugs, the DNA was mixed with GOs. The relative DOF of the samples were estimated by dividing DOF of the each sample by DOL of control, which was mixture of GO and UV-irradiated DNA in the absence of the tested drugs. *BP* benzophenone, *KP* ketoprofen, *IM* indomethacin, *NP* naproxen, *NR* norfloxacin

After validating the possibility of the detection of SDBs using GO, a parallel screening of SDB photosensitizers was carried out. The photosensitizing effect of the tested drugs was estimated with the relative DOF of the tested samples, DOF of each sample divided by DOF of control, which was UV-irradiated DNA in the absence of the drug such as three nonsteroidal anti-inflammatory drugs, ketoprofen (KP), naproxen (NP) and indomethacin (IM), norfloxacin (NR) and benzophenone (BP). IM and NR were known to have low photosensitizing efficiency [25–27]. Figure 4 shows the estimated photosensitized effect of the tested drugs. Among them, benzophenone (BP) resulted in the greatest DOF. The quantum yield of BP for singlet oxygen production was highest than that of other drugs [6]. Singlet oxygen is known to be a main species that oxidizes guanine and results in SDBs [6, 7, 28, 29]. Under the UV irradiation, a greater amount of reactive oxygen produced by BP, might break higher number of ssDNA than other photosensitizers tested in this study. Thus, GOs mixed with the DNA photosensitized by BP could adsorb relatively high numbers of DNA compared GO mixed with the DNA photosensitized with other drugs. Therefore, the highest DOF was observed from the DNA treated by BP than other drugs.

## Conclusion

In conclusion, herein GO was successfully applied to the screening of drugs for SDB-photosensitizing activity at the physiological condition in an operationally simple and high-throughput manner. In contrast to conventional methods, the GO based method described herein is ideal for the accelerated screening of a large number of drug candidates at the physiological condition because it does not require chemical or biochemical manipulation of the DNA. However, instability of AuNP in the presence of salt limits a practical application of the previously reported colorimetric method for evaluating phototoxicity of the drugs. Therefore, it could be expected that the presented method using GO will provide a feasible and practical approach for developing new drug candidates without phototoxicity.
